# Genetic association of fetal-hemoglobin levels in individuals with sickle cell disease in Tanzania maps to conserved regulatory elements within the *MYB* core enhancer

**DOI:** 10.1186/s12881-015-0148-3

**Published:** 2015-02-10

**Authors:** Siana N Mtatiro, Josephine Mgaya, Tarjinder Singh, Harvest Mariki, Helen Rooks, Deogratius Soka, Bruno Mmbando, Swee Lay Thein, Jeffrey C Barrett, Julie Makani, Sharon E Cox, Stephan Menzel

**Affiliations:** Muhimbili Wellcome Programme, Muhimbili University of Health and Allied Sciences, Dar-es-Salaam, Tanzania; Dar es Salaam University College of Education, Dar es Salaam, Tanzania; King’s College London, Molecular Haematology, Division of Cancer Studies, London, UK; Department of Human Genetics, Wellcome Trust Sanger Institute, Cambridge, UK; National Institute for Medical Research, Tanga Centre, Tanga, Tanzania; London School of Hygiene & Tropical Medicine, London, UK; Department of Haematology and Blood Transfusion, Muhimbili University of Health and Allied Sciences, PO Box 65001, Dar es Salaam, Tanzania; King’s College London, he James Black Centre, 125 Coldharbour Lane, London, SE5 9NU UK

## Abstract

**Background:**

Common genetic variants residing near upstream regulatory elements for *MYB*, the gene encoding transcription factor cMYB, promote the persistence of fetal hemoglobin (HbF) into adulthood. While they have no consequences in healthy individuals, high HbF levels have major clinical benefits in patients with sickle cell disease (SCD) or β thalassemia. Here, we present our detailed investigation of *HBS1L-MYB* intergenic polymorphism block 2 (*HMIP-2*), the central component of the complex quantitative-trait locus upstream of *MYB*, in 1,022 individuals with SCD in Tanzania.

**Methods:**

We have looked at 1022 individuals with HbSS or HbS/β^0^ in Tanzania. In order to achieve a detailed analysis of *HMIP-2*, we performed targeted genotyping for a total of 10 SNPs and extracted additional 528 SNPs information from a genome wide scan involving the same population. Using MACH, we utilized the existing YRI data from 1000 genomes to impute 54 SNPs situated within *HIMP-2*.

**Results:**

Seven HbF-increasing, low-frequency variants (β > 0.3, p < 10^−5^, f ≤ 0.05) were located in two partially-independent sub-loci, *HMIP-2A* and *HMIP-2B*. The spectrum of haplotypes carrying such alleles was diverse when compared to European and West African reference populations: we detected one such haplotype at sub-locus *HMIP-2A*, two at *HMIP-2B*, and a fourth including high-HbF alleles at both sub-loci (‘Eurasian’ haplotype clade). In the region of *HMIP-2A* a putative functional variant (a 3-bp indel) has been described previously, but no such candidate causative variant exists at *HMIP-2B*. Extending our dataset through imputation with 1000 Genomes, whole-genome-sequence data, we have mapped peak association at *HMIP-2B* to an 11-kb region around *rs9494145* and *rs9483788*, flanked by two conserved regulatory elements for *MYB*.

**Conclusions:**

Studies in populations from the African continent provide distinct opportunities for mapping disease-modifying genetic loci, especially for conditions that are highly prevalent there, such as SCD. Population-genetic characteristics of our cohort, such as ethnic diversity and the predominance of shorter, African-type haplotypes, can add to the power of such studies.

## Background

Sickle cell disease (SCD) is a hemoglobin disorder caused by the Glu6Val mutation in the β chain of adult hemoglobin. The resulting hemoglobin variant, HbS, is prone to polymerization, disrupting red blood cell shape, function and life span. SCD is prevalent in Sub-Saharan Africa, where it is a significant contributor to childhood mortality [1]. In Tanzania, 8,000-11,000 affected children are born annually [2]. The most common and severe forms of the disease are due to homozygosity for the mutation (HbSS) or compound heterozygosity with β^0^ thalassemia (HbS/β^0^thalassemia). Where newborn screening and prophylactic penicillin are available, childhood mortality due to SCD is significantly reduced, but patients nevertheless remain at risk for chronic complications and premature death. The disease is milder in those patients that carry significant amounts of fetal hemoglobin (HbF) in their circulating red blood cells [3]. Similar to healthy populations, HbF persistence in patients with SCD is partially genetically controlled, and three HbF quantitative-trait loci (QTLs) - *HBG2* [4,5], *BCL11A* [6,7] and *HBS1L-MYB* [8] - have been identified. Knowledge of the genetic factors underlying HbF persistence is helping to interpret the clinical variability of SCD and has led to the identification of novel molecular targets for the therapeutic reactivation of HbF.

*HBS1L-MYB* is unique among the HbF modifier loci because it has marked pleiotropic effects, i.e., in healthy individuals it affects general hematological parameters [9] as well as HbF. It has been postulated that changes in HbF levels caused by this locus are secondary to altered kinetics of erythropoiesis [10]. The locus consists of several linkage disequilibrium (LD) blocks of common variants, which affect erythroid traits independently [8]. The most effective of these, termed *HMIP-2* (*HBS1L-MYB* intergenic polymorphism, block 2) has been shown to influence disease severity in patients with SCD [11] and β thalassemia [8,12]. *HMIP-2* variants reside within the core enhancer for *MYB* [13], a key hematopoietic regulator gene [14]. It is divided further into sub-loci *HMIP-2A* and *-2B*, which provide independent HbF association in African populations, including SCD patients [11,15-18]. A 3-bp deletion (*rs66650371*) at *HMIP-2A* is suspected to directly cause HbF variability [19], but is independent of the trait association seen at *HMIP-2B*. Therefore, causative variants acting at *HMIP-2B* are still to be discovered.

To better define the HbF association signal at *HMIP-2B*, and to identify candidate variants for trait causation, we dissected *HMIP-2* and its effect on HbF persistence in a large SCD patient cohort from Tanzania. The Tanzanian population is well-suited to genetic fine-mapping studies, with a marked ethnic diversity [20,21] and the increased mapping resolution that is characteristic for African chromosomes [22,23].

## Methods

### Study subjects, sample collection and phenotyping

Only patients with Hb SS or HbS/β^0^ thalassemia genotype were included in this study. Enrollment of patients, diagnosis and confirmation of sickle phenotypes as well as the quantification of hemoglobin subtypes were performed as previously described [24]. Informed consent was obtained for each patient and ethical approval given by the Muhimbili University Research and Publications Committee (MU/RP/AEC/VOLX1/33). During follow-up clinics, a 2-ml blood sample was collected from non-transfused SCD patients (confirmed Hb SS genotype) who are not on hydroxyurea treatment. This study includes 1,022 individuals with HbF measured (by HPLC, Variant I, Biorad, Hercules, CA, USA) at the age of 5 years or older. The median age of the SCD population is 11 years; males and females are represented equally. HbF values vary significantly, with a median of 5.4% (of total hemoglobin).

### Genotyping

DNA was extracted from archived buffy coat using the Nucleon BACC II system (GE Healthcare, Little Chalfont, UK). Genotypes for 528 regional SNPs were extracted from a genome-wide SNP set generated at the Wellcome Trust Sanger Institute on the Human Omnichip 2.5 platform (Illumina, La Jolla, CA, USA), as described elsewhere [25]. Targeted genotyping was performed, adding ten markers with known trait association: *rs9376090*, *rs9399137*, *rs9402686*, *rs9389269* and *rs9494142* by TaqMan procedure [16], *rs9389268* and *rs9376091* by PCR product sequencing (amplification and sequencing: F: 5’-TGCTTCTGGCAGTGAATTAACCTTGT-3’, R: 5’-AGTTTGGTGCCAAAGGTAGCAGAT-3’), indels *rs66650371* and *rs11321816* by multiplex PCR fragment sizing (F1: 5’-GTTTGATGTTGCAGAAGAACAAAGC-3’ R1: 5’-VIC-TAAGTGTCTTCTGAGGGAACC-3’, F2: 5’-FAM-TCACCTTAAAAGGCGGTATTG-3’, R2: 5’-GTTT-AAGCACTTTGGCAAGCAT-3’) and *rs35786788* by SNaPshot procedure (F:5’-FAM-TCACCTTAAAAGGCGGTATTG-3’, R:5’-GTTT-AAGCACTTTGGCAAGCAT-3’, extension: 5’-ACTATATCTGTGCACAGAAATACAG-3’). All assays were performed under supplier-recommended conditions (Applied Biosystems, Foster City, CA, US), including the fragment sizing, which used the Taq Gold (Applied Biosystems) microsatellite genotyping protocol. Fragment sizes and SNaPshot products were evaluated by capillary electrophoresis (3130 Genetic analyzer, Applied Biosystems), with subsequent allele scoring using GeneMarker v1.95 (SoftGenetics, State College, PA, USA). Marker quality control consisted of Hardy-Weinberg equilibrium testing and call rate evaluation (cut off >80%). Imputation with MACH 1.0 [26,27] was used to fill in missing genotypes.

### Statistical analysis

Phased variant call files from the 1000 Genomes project [28] for the YRI population sample were accessed on 24/4/2013 (ftp://ftp.1000genomes.ebi.ac.uk/vol1/ftp/release/20110521/ALL.chr6.phase1_release_v3.20101123.snps_indels_svs.genotypes.vcf.gz) using the ‘Data Slicer’ tool at http://browser.1000genomes.org. Haplotype files were derived, purged of non-informative variants (monomorphic and singletons) and used to impute 54 non-genotyped variants in the target area, using MACH 1.0.16 [29,30].

GWAS data was processed with the PLINK software package (http://pngu.mgh.havard.edu/purcell/plink/). Test for genetic association with ln[%HbF], including conditional analysis, was performed with STATA v12 (Stata Corp, College Station, TX) using multiple linear regression with age and sex as covariates. Haplotype relative effects were estimated using multifactor ANOVA in R (http://www.r-projects.org/), correcting for pair-wise comparison using Tukey’s method, including age and sex as covariates.

## Results

### Genetic association with HbF in the *HBS1L-MYB* intergenic region

**Figure 1:**
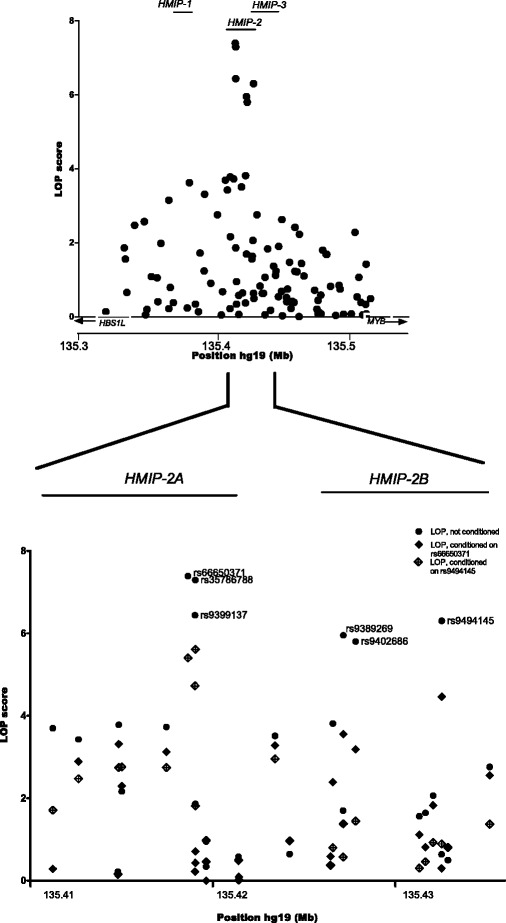
**Association of common genetic variation with HbF levels across the** ***HBS1L-MYB*** **intergenic region on chromosome 6q23.3.** Shown are LOP (−log_10_ [P-value]) scores for 1,022 patients, tested for association of ln[%HbF] with 109 common variants from a combination of Illumina Human Omnichip 2.5 data and PCR-based genotyping. **A**: Genetic association is present over the entire interval, but reaches genome-wide significance only at *HMIP-2*, whereas the other two LD blocks detected in Europeans (*HMIP-1* and *HMIP-3*,[8]) display only low-level association in our dataset. **B**: HbF association at *HMIP-2*. The six SNPs showing strong association are indicated. In addition to un-conditioned analysis (black dots), the presence two partially independent association signals (sub-loci *HMIP-2A* and *HMIP-32B*) is shown by conditioning analysis on *rs66650371* (tagging *HMIP-2A*, black diamonds) and *rs9494145* (tagging *HMIP-2B*, open diamonds, see also Table 1).

In 1,022 SCD patients, we scanned the *HBS1L*-*MYB* intergenic region (chr6:135,318,635-135,518,635, Figure 1 for genetic association with HbF levels (ln[%HbF]), evaluating 538 SNP (single nucleotide polymorphism) markers from a combination of genome-wide chip array and targeted genotyping. Confirming our own previous findings [9,15] and replicating what is seen across human populations [4,6-13], we detected trait association over the entire region, with the peak signal (P < 10^−7^) located at the *HMIP-2* locus (Figure 1, Table 1). Strong trait association at sub-locus *HMIP-2A* was detected with *rs66650371* (the 3-bp in/del proposed to be functional [19]), *rs35786788* and *rs9399137*, and at *HMIP-2B* with *rs9494145*, *rs9389269* and *rs9402686* (Figure 1, Table 1). The partial independence of the two sub-loci was confirmed in our dataset by conditional analysis (Table 1).

**Table 1:** **Association of** ***HMIP-2*** **variants with fetal-hemoglobin levels (ln[HbF%]) in Tanzanian patients with SCA**

**Marker**	**Sub-locus**	**Chr 6 position**	**allele change**	**MAF**	**β**	**P**	β*	P*	β**	P**	β***	P***
***rs9376090***		135,411,228	T → C	0.01	0.78	2.02x 10^−4^	0.17	0.52	0.51	0.02	−0.09	0.71
***rs41294858***		135,412,636	T → C	0.18	−0.16	3.74x 10^−4^	−0.14	1.28x 10^−3^	−0.13	3.35x 10^−3^	−0.12	6.07x 10^−3^
***rs55731938***		135,414,850	G → A	0.21	−0.16	1.65x 10^−4^	−0.15	4.85x 10^−4^	−0.13	1.79x 10^−3^	−0.13	2.68x 10^−3^
***rs7745098***		135,415,004	C → T	0.11	0.15	6.84x 10^−3^	0.15	5.04X 10^−3^	0.17	1.73x 10^−3^	0.17	1.68x 10^−3^
***rs76267242***		135,417,460	G → T	0.19	−0.16	1.87x 10^−4^	−0.15	7.52x 10^−4^	−0.14	1.78x 10^−3^	−0.13	3.58x 10^−3^
***rs66650371***	***HMIP-2A***	135,418,635	I → D	0.02	0.69	4.06 x 10^−8^			0.59	3.94x 10^−6^		
***rs9399137***	***HMIP-2A***	135,419,018	T → C	0.02	0.63	3.65 x 10^−7^	0.14	0.6	0.54	1.87x 10^−5^	0.13	0.61
***rs11321816***		135,419,038	I → D	0.14	0.12	1.36x 10^−2^	0.05	0.36	0.12	0.02	0.06	0.26
***rs35786788***	***HMIP-2A***	135,419,042	G → A	0.02	0.69	5.07 x 10^−8^	0.35	0.19	0.61	2.44x 10^−6^	0.37	0.16
***rs1074849***		135,423,412	G → A	0.13	−0.19	3.09x 10^−4^	−0.18	5.22x 10^−4^	−0.17	1.11x 10^−3^	−0.16	1.37x 10^−3^
***rs4895441***	***HMIP-2B***	135,426,573	A → G	0.05	0.30	1.53x 10^−4^	0.23	4.06x 10^−3^	0.13	0.16	0.09	0.35
***rs9389269***	***HMIP-2B***	135,427,159	T → C	0.03	0.48	1.11x 10^−6^	0.37	2.76x 10^−4^	0.27	0.04	0.16	0.22
***rs9402686***	***HMIP-2B***	135,427,817	G → A	0.03	0.49	1.57x 10^−6^	0.36	6.51x 10^−4^	0.27	0.04	0.14	0.28
***rs1411919***		135,432,061	A → G	0.34	−0.10	8.67x 10^−3^	−0.09	0.01	−0.06	0.12	−0.06	0.11
***rs9494145***	***HMIP-2B***	135,432,552	T → C	0.05	0.38	4.97 x 10^−7^	0.31	3.45x 10^−5^				
***rs2223385***		135,435,171	G → A	0.39	−0.11	1.74x 10^−3^	−0.11	2.77x 10^−3^	−0.08	0.04	−0.08	0.04
***rs1320963***		135,443,212	A → G	0.33	0.09	1.44x 10^−2^	0.07	0.05	0.03	0.44	0.02	0.54
***rs1569534***		135,451,580	C → T	0.15	−0.12	1.25x 10^−2^	−0.11	0.02	−0.10	0.04	−0.09	0.06
***rs6929404***		135,454,027	C → A	0.49	0.11	2.35x 10^−3^	0.09	6.64x 10^−3^	0.06	0.08	0.06	0.09

linear regression analysis: *conditioned for *rs66650371*, **conditioned on *rs9494145*, ***conditioned on *rs66650371*and *rs9494145*. (Residual association extends upstream of *HMIP-2* and overlaps with the previously reported (Thein et al., PNAS 2007) *HMIP-1* signal.).

The chromosomal position is given in hg19 coordinates, derived using UCSC Genome Browser version February 2009.

MAF: Minor allele frequency of the patient cohort; I: inserted allele, D: deleted allele (Δ ‘TAY’). ß, the un-standardized regression co-efficient is given as a measure of the effect of the allele change on ln[%HbF].

Shown are all directly genotyped markers with nominally significant association at p < 0.01.

### African-specific trait association at *HMIP-2B*

**Figure 2:**
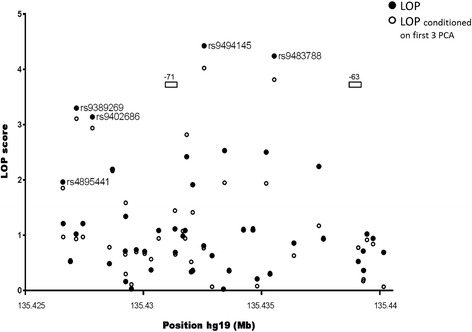
**African-specific association with HbF at** ***HMIP-2B*** **in Tanzanian SCD patients.** Association analysis was performed with the same individuals as in Figure 1, but 15 patients with Eurasian-type high-HbF haplotypes (‘A – B’, carrying the ancestry-informative allele *rs1376090*-C) were excluded. Data for 54 markers imputed from 1000 Genomes YRI (Yoruba, Ibadan, Nigeria) sequence were added to the analysis. While the exclusion of selected individuals resulted in a weaker overall association signal, the potential to map African-specific variants at higher resolution was considered more important at this stage. Association scores are shown; unconditioned (black dots) and conditioned on the first three principal components derived from genome-wide SNP data (open circles) [25]. Shown also is the location of the conserved *MYB* upstream regulatory elements −71 and −63 [13].

In contrast to *HMIP-2A*, a good candidate variant for causing the HbF association at *HMIP-2B* has so far not been identified. We therefore studied HbF association at this sub-locus in more detail. We included additional variants from 1000 Genomes project sequence data [28] through imputation with YRI (Yoruba, West African) haplotypes, which are well-matched with our population regarding the allele frequencies of regional markers [18]. The YRI dataset harbors 51 polymorphic variants within the *HMIP-2B* region (chr6:135,426,573-135,435,501), and another 17 such variants in an additional 5-kb fragment we decided to include on the 3’ side of the locus, adjacent to *MYB*. 15 patients carrying European-type high-HbF haplotypes (tagged by rs1376090 [18]) were excluded from imputation and analysis to make full use of the increased mapping resolution of the shorter African haplotypes, with the goal to localize the QTL more precisely. Association analysis subsequent to imputation identified an additional SNP with strong HbF association, *rs9483788* (Figure 2). The area of significant association (p < 0.01) thus extends between chr6:135,427,000 and 135,438,000, with the peak between MYB enhancer elements −71 and −63 (Figure 2).

### *HMIP-2* haplotypes

To dissect the haplotype architecture underlying the trait association pattern at *HMIP-2*, we phase-aligned genotypes for the seven strongly-associated markers. To relate our data to findings in other populations, we also included *rs9376090*, which tags European and Asian high-HbF alleles, and *rs4895441*, which is part of the *HMIP-2B* sub-locus in other SCD patient populations.

**Figure 3:**
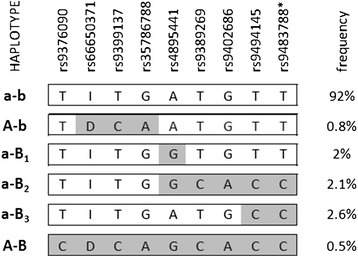
***HMIP-2*** **haplotypes detected in Tanzanian SCD patients.** Nine critical variants at *HMIP-2* were used to investigate haplotypes present at the locus (alignment by Phase v. 2.1). Haplotypes were assigned to the principal clades described previously [18], dependent on whether they contain HbF-increasing alleles (shaded in gray) at *HMIP-2A* (capital ‘A’) or *HMIP-2B* (capital ‘B’). a-b: ancestral haplotype present in all human populations, composed entirely of low-HbF associated alleles; A-b: HbF increasing alleles at *HMIP-2A*, but lacking the European/Asian-specific allele *rs9376090*-C; a-B: a-B_1_: one HbF-increasing allele at *HMIP-2B*, *rs4895441*-G, a-B_2_: HbF-increasing alleles across *HMIP-2B*; a-B_3_: two HbF-increasing alleles at *HMIP-2B*, *rs9494145*-C and rs9483788-C, A-B: Eurasian haplotype, HbF-increasing alleles across all of *HMIP-2*; Rare haplotypes (frequency < 0.5%) are not shown. *imputed

**Figure 4:**
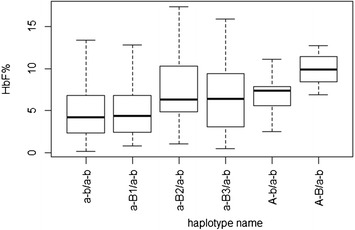
**HbF levels according to** ***HMIP-2*** **haplotype combination.** %HbF of total hemoglobin was measured by HPLC in patients carrying one copy of the ancestral haplotype, with diverse haplotypes on the sister chromosome. Boxes show median and inter-quartile range. Relative effects of these haplotypes are shown in Table 2.

The most prevalent haplotype carried the reference (low-HbF) allele in all positions, representing the global ancestral situation, universally associated with low HbF levels [18]. The other haplotypes contained high-HbF associated alleles in at least one position (Figure 3). A’Eurasian-type’ haplotype [18] (high-HbF alleles at all seven positions, including the ancestry-informative marker *rs9376090*) was detected at low frequency, but with high HbF levels (9.3%, median, Figure 4).

The remaining haplotypes belonged to two minor clades more common in African populations, one with HbF-promoting alleles at *HMIP-2A* only (termed clade ‘A - b’), and one with HbF-promoting alleles at *HMIP-2B* only (clade ‘a - B’). In Tanzanian patients, ‘A - b’ was represented by a single haplotype, whereas the ‘a - B’ clade was more diverse (haplotypes ‘a - B_1_’, ‘a – B_2_’, ‘a – B_3_’). ‘A - b’, ‘a - B_2_’ and ‘a - B_3_’ haplotypes all significantly boost HbF levels, compared to the ancestral haplotype ‘a - b’ (Table 2), while ‘a - B_1_’ does not. ‘a - B_1_’ contains only a single non-ancestral allele, *rs4895441*-G, which is not strongly HbF-associated in Tanzanian patients. ‘a - B_3_’, which contains two high-HbF associated alleles, *rs9494145*-C and *rs9483788*-C, significantly increases HbF levels over the ancestral haplotype. A stronger effect is detected when all four HbF-boosting alleles, *rs9389269*-C, *rs9402686*-A, *rs9494145*-C and *rs9483788*-C, are present (in ‘a - B_2_’, Table 2).

**Table 2:** **Relative effects of** ***HMIP-2*** **haplotypes on the ln[HbF%] trait**

**Compared haplotypes**	**Difference**	**lower**	**upper**	**p adjusted**
a-B_1_ vs. a-b	0.029	−0.35	0.40	1.00
**a-B**_**2**_ **vs. a-b**	**0.431**	**0.08**	**0.78**	**7 x 10** ^**−3**^
**a-B**_**3**_ **vs. a-b**	**0.332**	**0.01**	**0.66**	**0.04**
**A-b vs. a-b**	**0.636**	**0.10**	**1.18**	**0.01**
A-B vs. a-b	0.638	−0.10	1.38	0.13
a-B_1_ vs. a-B_2_	−0.401	−0.10	0.90	0.20
a-B_1_ vs. a-B_3_	−0.303	−0.79	0.18	0.47
a-B_2_ vs. a-B_3_	0.098	−0.37	0.56	0.99
a-B_1_ vs. A-b	−0.607	−1.25	0.04	0.08
a-B_2_ vs. A-b	−0.205	−0.84	0.43	0.94
a-B_3_ vs. A-b	−0.304	−0.92	0.32	0.73
A-B vs. A-b	0.002	−0.91	0.91	1.00
a-B_1_ vs. A-B	−0.609	−1.43	0.21	0.28
a-B_2_ vs. A-B	−0.208	−1.02	0.60	0.98
a-B_3_ vs. A-B	−0.306	−1.10	0.49	0.88

“Difference” represents the difference in effect size between the two haplotypes on log-transformed HbF levels; negative difference values indicates that the effect size of the second haplotype is bigger than that of the first. ‘lower’ and ‘upper’ represent boundaries for family-wise 95% confidence intervals. ‘p adjusted’ is the P-value adjusted for age, sex as well as multiple testing.

Three haplotypes (shown in bold) have significant HbF-boosting effects at the 0.05 level.

## Discussion

Performing trait-association for SNPs across the *HBS1L-MYB* intergenic region on chromosome 6q24.3 in SCD patients from Tanzania, we detected significant association with HbF levels at *HMIP-2*, a globally-prevalent HbF QTL [7-9,11,15,17,19,31-35] residing within the *MYB* enhancer region [13]. Some of these variants have been also associated with white blood counts, mean cell volume and mean cell hemoglobin in our population [36]. Our interest was focused on sub-locus *HMIP-2B*, where a causative variant has not yet been identified. After excluding patients with longer, ‘Eurasian’-type [18] high-HbF haplotypes and including imputed variants from the YRI (Yoruba, 1000 Genomes sequence data) population, we determined the most likely map location of *HMIP-2B* as an 11-kb segment including the enhancer core element −71 and the interval between elements −71 and −63 (Figure 2), where peak association (*rs9494145*, *rs9483788*) was detected.

The two HbF-boosting haplotypes underlying this association peak, ‘a – B_2_’ and ‘a – B_3_’, share *rs9494145*-C and *rs9483788*-C (Figure 3). ‘a - B_2_’, which contains all four HbF-boosting alleles (*rs9389269*-C, *rs9402686*-A, *rs9494145*-C and *rs9483788*-C), has the stronger effect of the two. This means that none of the four strongly trait-associated SNPs detected at *HMIP-2B* in Tanzanian patients appears to fulfill the conditions for being the singular causative variant, i.e. both, being necessary to show a significant effect and sufficient to produce the maximum genetic effect originating from this sub-locus. Thus, additional variants, not present in the 1000 Genomes dataset, might contribute to trait variability.

Long, ‘Eurasian-type’ (with high-HbF associated alleles across all of *HMIP-2* [18]), high-HbF haplotypes were present in the patient cohort at a low frequency. These haplotypes are tagged by the ancestry-informative allele rs9376090-C (Figure 3). 24% of individuals with such haplotypes reported Arabic parental ethnicity, compared to 2% in the general cohort. The high HbF levels we observed in such patients (a median of 9.3% in ‘A-B’/’a-b’ heterozygotes, Figure 4) are likely due to the presence of the 3-bp deleted allele at *HMIP-2A* and possibly another functional allele at *HMIP-2B*. Population stratification might also contribute to higher levels of HbF: Arab/Indian sickle mutation haplotypes on chromosome 11 are known to result in milder disease and high HbF levels [37].

We also observed a residual association after conditioning on *HMIP-2A* and *HMIP-2B*. We suspect that these are part of a group of linked SNPs that overlaps the physical location of *HMIP-1*, a HbF QTL detected upstream of *HMIP-2* (Figure 1A) in the European population [8]. However we didn’t feel we have the power to investigate this further with the present dataset.

## Conclusions

We have localized *HMIP-2B*, a QTL for fetal-hemoglobin persistence, to an 11-kb region within the core enhancer for *MYB*. So far, we have not identified a likely functional variant within or at this locus. Further studies will involve extended sequence analysis in groups of patients carrying a-B_2_ and a-B_3_ haplotypes.
